# Decoding executed and imagined grasping movements from distributed non-motor brain areas using a Riemannian decoder

**DOI:** 10.3389/fnins.2023.1283491

**Published:** 2023-11-23

**Authors:** Maarten C. Ottenhoff, Maxime Verwoert, Sophocles Goulis, Albert J. Colon, Louis Wagner, Simon Tousseyn, Johannes P. van Dijk, Pieter L. Kubben, Christian Herff

**Affiliations:** ^1^Department of Neurosurgery, Maastricht University, Maastricht, Netherlands; ^2^Academic Center for Epileptology, Kempenhaeghe, Heeze, Netherlands; ^3^Department of Orthodontics, Ulm University, Ulm, Germany; ^4^Department of Electrical Engineering, Eindhoven University of Technology, Eindhoven, Netherlands

**Keywords:** motor decoding, low-dimensional representation, distributed recordings, Riemannian geometry, brain-computer interfaces

## Abstract

Using brain activity directly as input for assistive tool control can circumventmuscular dysfunction and increase functional independence for physically impaired people. The motor cortex is commonly targeted for recordings, while growing evidence shows that there exists decodable movement-related neural activity outside of the motor cortex. Several decoding studies demonstrated significant decoding from distributed areas separately. Here, we combine information from all recorded non-motor brain areas and decode executed and imagined movements using a Riemannian decoder. We recorded neural activity from 8 epilepsy patients implanted with stereotactic-electroencephalographic electrodes (sEEG), while they performed an executed and imagined grasping tasks. Before decoding, we excluded all contacts in or adjacent to the central sulcus. The decoder extracts a low-dimensional representation of varying number of components, and classified move/no-move using a minimum-distance-to-geometric-mean Riemannian classifier. We show that executed and imagined movements can be decoded from distributed non-motor brain areas using a Riemannian decoder, reaching an area under the receiver operator characteristic of 0.83 ± 0.11. Furthermore, we highlight the distributedness of the movement-related neural activity, as no single brain area is the main driver of performance. Our decoding results demonstrate a first application of a Riemannian decoder on sEEG data and show that it is able to decode from distributed brain-wide recordings outside of the motor cortex. This brief report highlights the perspective to explore motor-related neural activity beyond the motor cortex, as many areas contain decodable information.

## Introduction

Motor neuron diseases, aging-related diseases and accidents can lead to losing a part of or complete muscle control: in the Netherlands alone, 415.000 people are experiencing severe physical disability (2011) (de Klerk et al., 2012; Jongh et al., 2021). A main predictor of their life satisfaction is their functional independence (Scott Richards et al., 1999; van Leeuwen et al., 2011), which could be regained with appropriate assistive tools. An intuitive way to increase functional independence again is to circumvent muscular dysfunction by using brain activity directly as input for control of assistive tools (Daly and Huggins, 2015; Gilja et al., 2015). To achieve this, decoding studies target the primary motor cortex to capture movement-related neural activity (Pandarinath et al., 2017; Flesher et al., 2021; Moses et al., 2021; Chaudhary et al., 2022). For example, implantations of microelectrode arrays (MEA) in the hand-knob area of the human primary motor cortex have resulted in state-of-the-art decoders that can decode imagined handwriting at speeds comparable to regular smartphone typing (Willett et al., 2020). However, the motor-related activity from the motor cortex may not capture the full extent of the motor system (Gallego et al., 2022), as descending motor neurons and concrete motor commands originate from other brain areas than the primary motor cortex as well (Strick et al., 2021). Furthermore, motor-related activity is more widespread than previously thought (Steinmetz et al., 2019).

Accordingly, multiple invasive studies reported decoding of motor-related activity outside of the motor cortex in humans, and found significant decoding results from multiple cortical and sub-cortical areas, such as the ventral premotor cortex (Wandelt et al., 2022), posterior parietal cortex (Andersen et al., 2019; Wang et al., 2020; Li et al., 2022), somatosensory cortex (Wandelt et al., 2022), supramarginal gyrus (Li et al., 2022; Wandelt et al., 2022), temporal areas (Breault et al., 2019), insula (Breault et al., 2019; Li et al., 2022), hippocampus (Breault et al., 2019; Li et al., 2022), basal ganglia (Mamun et al., 2015) and subthalamic nucleus (Shah et al., 2018). So far, all non-primary motor decoding studies show promising results by decoding significantly above chance from many areas individually. Leveraging all brain-wide information by including all channels may increase decoding power.

However, including all channels increases the risk of a poor decoder fit. The increased dimensionality may leave too little data to for the decoder to train on. Furthermore, including neural activity from brain wide areas might include more channels that do not hold any movement-related information, decreasing the signal-to-noise ratio. To address this dimensionality issue, techniques like principal component analysis can be used to acquire a low-dimensional representation of the neural data (Gallego et al., 2018). Furthermore, techniques such as Riemannian decoders (Congedo et al., 2017) used in surface EEG, known for its low signal to noise ratio, may be applicable to sEEG data as well.

**Figure 1:**
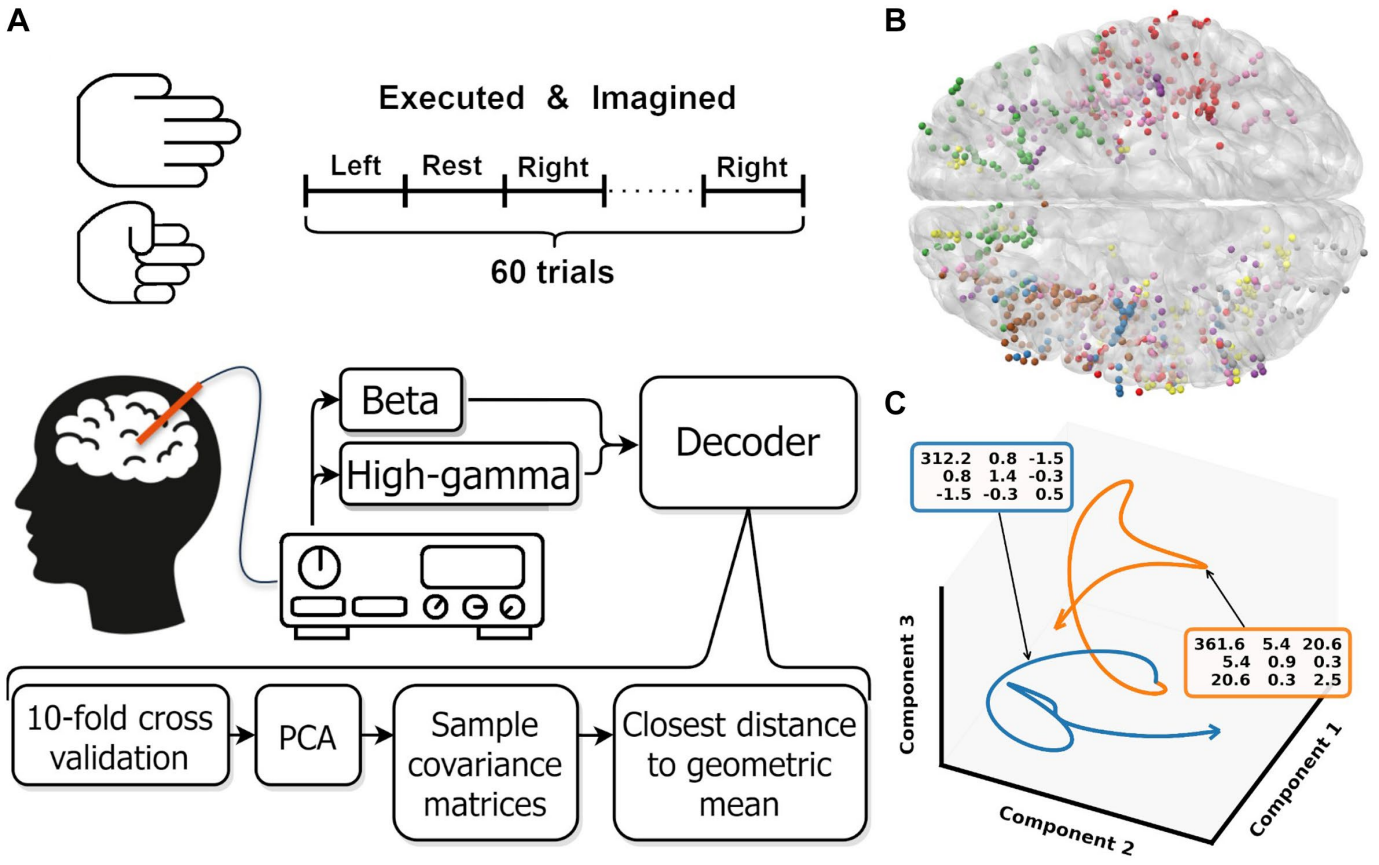
**(A)** Overview experimental protocol. **(B)** Contact locations of all participants warped onto an average brain. Each color represents contacts from one participant. **(C)** Low dimensional representation of the average movement (blue) and rest (orange) trial for one participant. For both trajectories, the covariance matrix of the first three components is shown in the colored boxes. These covariance matrices are used as input for the Riemannian decoder. The trajectories shown are smoothed by a low pass filter, the unsmoothed trajectories are shown in Supplementary Figure S1. Note that the trajectories are clearly separated in the space spanned by the first three components.

**Figure 2:**
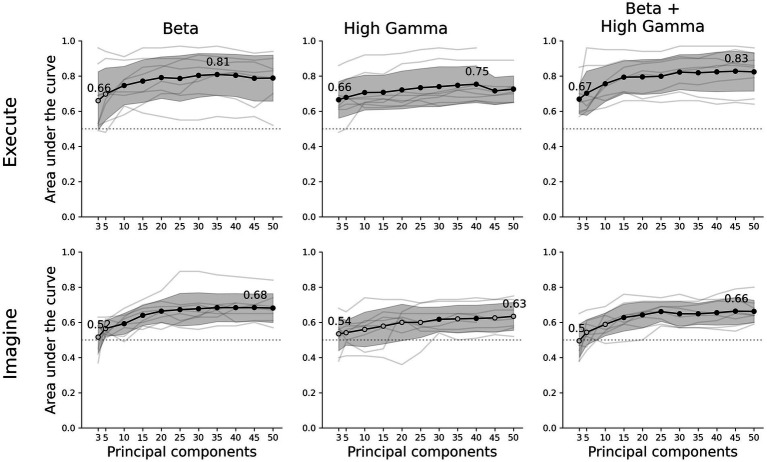
Decoder performance for different movement tasks, frequency features and number of components. The rows show the results of the executed or imagined movement task and the columns each frequency feature set used as input for the decoder. The x-axis depicts the amount of principal components extracted from the data set and the y-axis the AUC score. The light grey lines show the individual average scores over all folds per participant and the black circles are the average scores for each number of components. A filled black circle represents an average score that is significantly above chance (corrected for multiple testing), whereas an empty circle is not significant. The grey shaded area shows the standard deviation over participants and the dotted line the chance level (0.5 AUC).

Here, we expand from decoding movement from individual non-motor brain areas to including all available information. We capture whole-brain activity by recording data from stereotactic-electroencephalographic (sEEG) electrodes implanted in epilepsy patients. Combined over participants these electrodes cover the whole brain and provide a high-spatial and temporal resolution (Herff et al., 2020). To ensure we only include data from non-primary motor areas, we remove all electrode contacts around the central sulcus bilaterally. We reduce the dimensionality of the signal into a low-dimensional representation and apply a Riemannian decoder that directly classifies based on the covariance matrix of this representation (Figure 1A). We show significant above chance performance for both executed and imagined movements for nearly all number of principal components (Figure 2), without the need for areas surrounding the central sulcus.

## Methods

### Participants

Eight participants were included in this work (age 35.8 ± 14.2 years, mean ± SD; 5 male, 3 female, Supplementary Table S1). All participants are refractory epilepsy patients undergoing presurgical assessment for resection surgery. They were implanted with sEEG electrodes for two to three weeks to monitor seizures and identify the epileptogenic zone. The electrode placement and trajectories were determined solely based on their clinical needs. Participants were implanted with 5 to 14 electrodes containing 42 to 125 recordable contacts.

### Tasks

Each participant was asked to continuously open and close their hand for 3 s per trial follow by a 3 s rest period. 30 trials were cued per hand, resulting in 60 move and 60 rest trials (Figure 1A). The stimuli were presented in random order on a laptop screen that was resting on the participants lap or on a table in front. We ran the protocol for executed and imagined grasping movements. Participants were instructed to move only their hands and to keep the rest of their body still during executed grasping. For imagined movements, the participants were asked to remain completely still, and the experimenter visually checked if the participants adhered to the instruction. We did not use stricter or more objective methods like electromyography (EMG) to measure any micro-movements or increased muscle tension (Sburlea and Muller-Putz, 2018). In our experience, participants often find it challenging to imagine movements. Therefore, we always preceded the imagined grasping task with the executed grasping task to provide the participant with a fresh memory of the kinematic and proprioceptive sensation of a grasping movement. We assumed it was easier for our participant to recall a mental image of the grasping movement, helping them to perform the imagery task as good as possible. Additionally, the experimenter briefly introduced two potential imagery strategies: kinesthetic or visual (Hanakawa, 2016), but the participants were free to use any strategy that they thought was most effective for them.

### Ethical approval

The experimental protocol was approved by the institutional review board of Maastricht University and Epilepsy Center Kempenhaeghe (METC 2018-0451). All experiments were in accordance with the local guidelines and regulations and under supervision of experienced healthcare staff. All participants joined the study voluntarily and gave written informed consent.

### Data recording

Neural activity was recorded by platinum-iridium sEEG electrodes (Microdeep intracerebral electrodes; Dixi Medical, Beçanson, France) using two stacked 64-channel Micromed SD LTM Amplifiers (Micromed S.p.A., Treviso, Italy). The electrodes are 0.8 mm in diameter and contain 5 to 18 contacts. The contacts are 2 mm in length, have a 1.5 mm intercontact distance, and are referenced to a white matter electrode that did not show epileptic activity, visually determined by the epileptologist. All recordings and stimuli were synchronized using LabStreamingLayer (Kothe, 2014). For clarity, throughout this work we refer to ‘electrode’ as the implanted shaft and ‘contact’ for each location on each electrode where activity is measured.

### Imaging

The anatomical locations for each contact were determined using the img pipe Python package (Hamilton et al., 2017) and parcellation based on the Destrieux atlas (Destrieux et al., 2010). To do so, we coregistered a pre-implantation anatomical T1-weighted MRI scan, parcellated using Freesurfer,^1^ and a post-implantation CT scan. For visualization purposes, the electrodes were warped to average brain from the CVS average-35 atlas in MNI152 space.

To remove motor cortical areas we excluded all contacts of which the determined anatomical label contained the word ‘motor’ or ‘central’ (Supplementary Data 1). This was a strict exclusion of contacts, meaning that contacts in white matter close to the central sulcus and primary (sensori-)motor cortex are removed as well. Note that the white matter anatomical labels in the Destrieux atlas are based on proximity to labeled grey matter area, introducing some uncertainty of the exact location.

### Electrode coverage

In total, 956 contacts on 82 electrodes were implanted in our participants, with electrodes containing a minimum of 5 and a maximum of 18 contacts per electrode (Figure 1B). All contacts across participants covered 59 unique grey matter areas with 448 contacts, where the superior insular sulcus is covered the most (*n* = 25) followed by the superior temporal sulcus (*n* = 23) and the middle frontal gyrus (*n* = 23). The remaining contacts are located in white matter (*n* = 408) or unknown areas (*n* = 100). Unknown areas are areas that could not be identified due to various technical reasons. See Supplementary Figure S2 for a graphical overview of all areas. Because of a limited number of channels (*n* = 128) that can be recorded by the amplifiers, not all contact could be recorded, reducing the total amount of recorded contacts by 71 (Supplementary Table S1). The selection of which contacts should be included was made by the epileptologist for clinical reasons. The amount of recorded contacts left after motor and noise removal are shown in Supplementary Table S1.

### Preprocessing

First, we removed all contact in areas in or adjacent to the central sulcus (Supplementary data 2 for a complete list of removed labels). Then, we evaluated the signal quality of each contact by assessing excessive noise. First, contacts were flagged if the 50 Hz frequency band power exceeded two times the interquartile range of the signal. Additionally, contacts with a *z*-scored log square mean value that was significantly higher (*p* < 0.05, assuming normal distribution) than the values in other contacts were flagged for abnormal amplitude (Supplementary Table S1). The remaining contacts were detrended, demeaned and band-stop filtered for 50 Hz line noise its and harmonics up to and including 200 Hz, using a finite impulse response filter implemented in the MNE python package (Gramfort et al., 2014). Then, we extracted beta (12–30 Hz) and high-gamma (55–90 Hz) envelope by taking the absolute of the Hilbert transform on the band-passed filtered signal. These frequency bands are chosen as they are known to be movement related and have shown to be effective in decoding studies (Combrisson et al., 2017; Shah et al., 2017; Moses et al., 2021; Miller et al., 2022). After preprocessing, the data was split into trials. Left and right hand movement trials were combined into a single movement class.

### Decoder

A decoder was trained and tested for [3, 5, 10,…, 50] principle components and beta, high-gamma and beta + high-gamma bands. One participant had less than 50 contacts and could therefore not be evaluated with 50 components. Each component and band combination was trained and evaluated as follows: first, the data was split using 10-fold cross validation. On the training data, the data was standardized over all included trials per fold and a principal component analysis was performed. The learned transformation was subsequently used to transform the training and test fold to the specific amount of principal components. After transformation into the components space, the sample covariance matrix for each trial was calculated and regularized by the Ledoit and Wolf (2004). Figure 1C shows the average behavior per class for one participant. The covariance matrices are used as input for the Riemannian decoder. Then, the geometric mean per class was calculated based on the Kullback–Leibler divergence. Trials were then classified by selecting the class with the shortest distance to class geometric mean. For the calculations, we used the pyRiemann implementation (Barachant, 2015).

### Evaluation

We evaluated the decoder by the area under the receiver operator characteristics (AUC). We tested statistical significance against chance level (mean *AUC* = 0.5) using a one sample t-test and corrected for multiple testing using Bonferroni correction. For the control analysis for motor cortical areas, we used a Wilcoxon signed rank-test (Bonferroni corrected, *n* = 66, Supplementary Table S2) to compare the difference in performance with and without motor cortical areas. We compared the Riemannian decoder with a common spatial pattern (Koles et al., 1990) and linear discriminant analysis (CSP-LDA) decoder. Covariance matrices estimated during the CSP analysis were regularized using Ledoit-Wolf regularization (Ledoit and Wolf, 2004). After spatial filtering, the average power for each CSP was calculated. We used the MNE implementation of CSP (Gramfort et al., 2014).

## Results

Our classifier was able to decode executed movements from rest periods significantly above chance for all number of principal components and frequency features, except beta using 3 or 5 components. The highest performance was achieved by combining beta and high-gamma activity with 45 principal components (0.83 ± 0.11 AUC ± SD, Figure 2). Using only beta or high-gamma reached 0.81 ± 0.12 and 0.75 ± 0.10, respectively. For the imagined movement task, the decoder reached above chance performance for most number of components for both beta and beta + high-gamma. However, including only high-gamma produced barely any significant decoding results. Lower number of principal components did not reach above chance decoding, specifically: 3 and 5 in beta, 3, 5, or 10 in beta + high-gamma. Overall, decoding imagined movements yielded lower performance than decoding executed movements. The maximum performance for imagined movements using beta, high-gamma or beta + high-gamma was 0.68 ± 0.08, 0.63 ± 0.08 and 0.66 ± 0.06, respectively. The decoder performed comparable to a CSP-LDA decoder, where the latter performed better with fewer CSPs (<±25) and the former with more components (>±25, Supplementary Figure S2).

For high-gamma and beta + high-gamma in executed movements, the decoder was able to decode significantly above chance for all number of principal components. For beta, at least 10 were required. In the imagined tasks, at least 10 components were required as well for beta power. For high-gamma however, only 30 and 40 components were sufficient. Combining both beta and high-gamma showed that at least 15 components were required. Overall, it seems that 10 to 15 components are sufficient to reliably decode movement in both tasks. Increasing the amount of components gradually increases performance, where the maximum performance is between 35 to 50 components. However, the increase in performance per extra component decreases as more components are added, and stabilizes at about 25 components.

**Figure 3:**
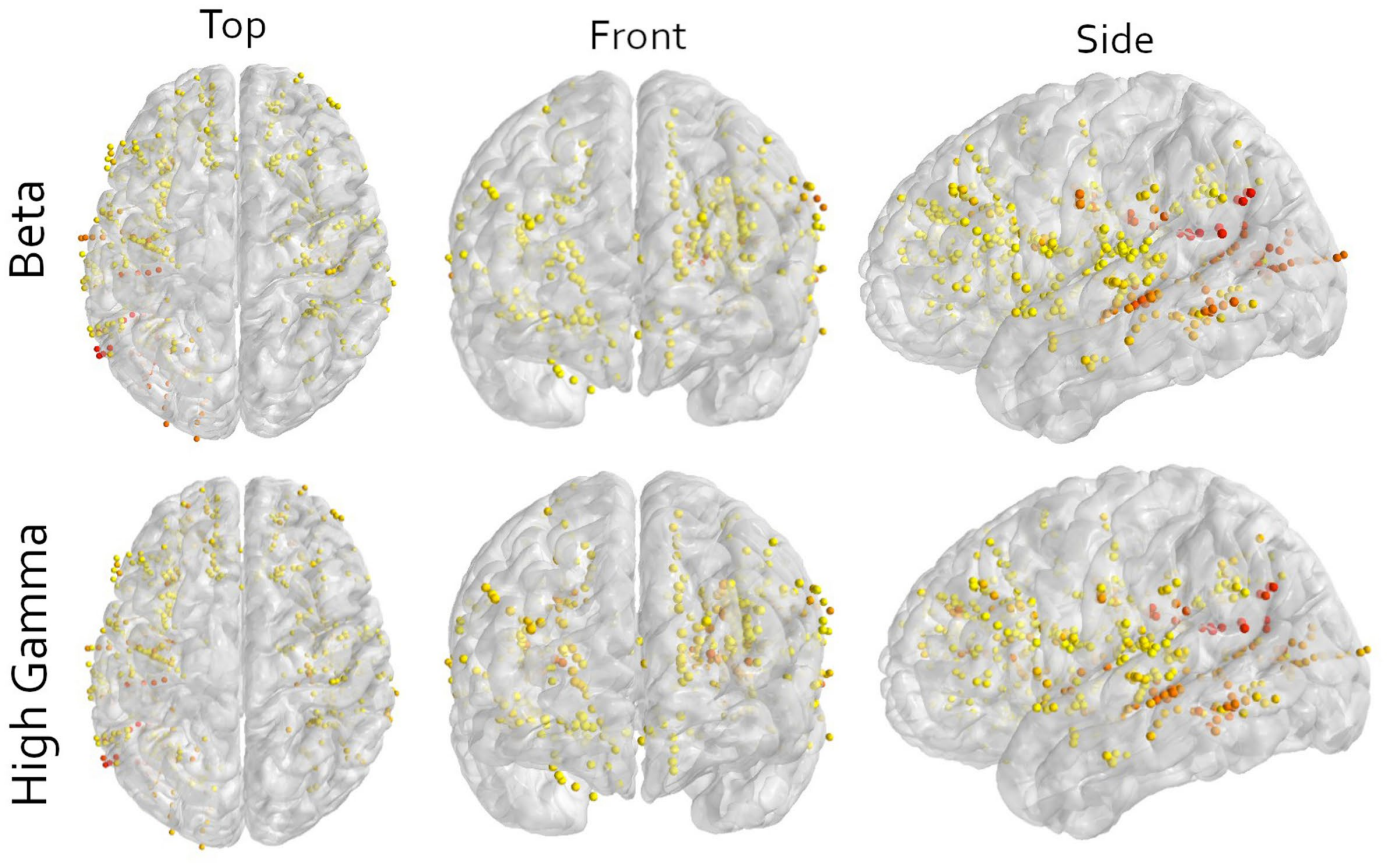
Multi-angle view of all contacts of all participants warped to an average brain for either the beta or high-gamma frequency in the imagined movement task. All contacts in motor cortical areas are excluded. The color indicates the contribution of that contact to the first principal component, scaled to the explained variance of that component. Yellow means low contribution and red mean high contribution. The image shows that orange and red colors are not bound to a specific area, illustrating the wide distribution of information. Note that here only the contributions to the first principal components are shown and that sufficient decoding requires at least 3 components (Figure 2). Furthermore, to visualize all contacts, the electrodes are non-linearly warped onto an average brain. This may result in contacts appearing to be in or around the central sulcus. This is an unpreventable visual artifact, and all locations are determined in the patient’s native space.

In this work, we included all available contacts in the decoding pipeline, except those around the central sulcus. When visualizing the contribution of each electrode to the first principal components, a distributed pattern is visible (Figure 3, red and yellow for high and low contribution, respectively). While there are a few regions contributing more to the first component than others, mostly posterior areas, it seems like motor-related information is distributed throughout the brain. Specifically considering that at least 3 to 10 components are required for above chance decoding.

## Discussion

Here, we demonstrate that a Riemannian decoder is able to decode both executed and imagined movements using a low-dimensional representation from distributed brain-wide recordings. Furthermore, we show that non-motor brain areas contain sufficient information for our decoder to predict movement significantly above chance.

Our results support the notion that movement-related activity is widespread throughout the brain and that extracting a lower-dimensional representation is effective to capture this distributed activity (Stringer et al., 2019; Gallego et al., 2022). So far, studies decoding motor-related activity from distributed recordings have investigated contributions per contact or grouped cortical areas (Andersen et al., 2019; Li et al., 2022; Wandelt et al., 2022). Here, we expand to include neural activity from all brain regions, excluding those surrounding the central sulcus. Using this approach, we were able to decode significantly above chance for almost all participants. Specifically, when using beta & high-gamma as input power bands, we were able to decode above chance, regardless of electrode configuration (Figure 2).

Although our decoder was able to predict movements, the used methods include any signal that is relevant for the classification task, and no selection is made based on a mechanistic presumption. Thus, the relevant information may also include any other motor related signal, like motor planning, sequencing or decision-making, as well as non-motor information such as attention, stimulus processing, stimulus comprehension or spatial information. The used paradigm does not allow us to make an inference of the contents of the neural signals. Nonetheless, looking at the contributions per electrode indicates that it is not a single area driving the performance, but the combination of many different non-motor areas (Figure 3). This is supported by the observation that multiple different electrode configurations resulted in above chance decoding (Figures 1B, 2).

The performance of our Riemannian decoder demonstrates that this type of decoder is applicable to the distributed recordings of sEEG. The presented pipeline is simple and near non-parametric. While there are multiple variations of Riemannian decoders (Yger et al., 2017), the only parameter we choose was the distance metric [Kullback–Leibler, based on Chevallier et al. (2021)], and the number of principal components. When using Riemannian decoders the dimensionality should preferably to be low. During training, the decoder calculates the geometric mean between all sample covariance matrices per class, which is an optimization problem that scales exponentially with increased dimensions.

Furthermore, using a low-dimensional representation combines information from all contacts, which separately might not have enough information for sufficient decoding. Since the information is distributed throughout the brain, the loss of single contacts likely only has a minor influence on overall decoding performance. This is especially useful in the eventual target population, were neurodegenerative diseases might cause specific brain areas to stop contributing information, or electrode degradation can decrease the recorded activity from a contact.

### Limitations

All our participants are diagnosed with refractory epilepsy, a condition of which it is unclear how it influences our decoding results. During the monitoring phase in which we perform our measurements, our participants are expected to have as much seizures as possible, albeit no seizures occurred during one of the experimental sessions. After a few days of settling in the monitoring center, medication is reduced and eventually the participants are stimulated in various forms to elicit seizures. Therefore, participants often feel drowsy and experience post-ictal discharges. We try to reduce influences as much as possible by visiting as early in their treatment as possible, but we are dependent on the clinical schedule of the patient. Lastly, our decoder is evaluated on a trial-based paradigm, and thus cannot be applied in real-time decoding applications in its current form.

## Conclusion

Both executed and imagined movements can be decoded from distributed non-motor brain areas using a lower dimensional representation from sEEG electrodes. We demonstrate that a Riemannian decoder captures relevant movement-related information that is spread throughout the brain, which hold enough information to predict movement. Future work may focus on optimizing Riemannian methods on distributed data, and application in an online paradigm.

## Data availability statement

The datasets presented in this study can be found at https://osf.io/xw386, and the accompanying code at https://github.com/mottenhoff/distributed_motor_decoding.

## Ethics statement

The studies involving humans were approved by the Institutional review board of Maastricht University and Epilepsy Center Kempenhaeghe. The studies were conducted in accordance with the local legislation and institutional requirements. Written informed consent for participation in this study was provided by the participants’ legal guardians/next of kin. Written informed consent was obtained from the individual(s), and minor(s)’ legal guardian/next of kin, for the publication of any potentially identifiable images or data included in this article.

## Author contributions

MO: Conceptualization, Investigation, Software, Writing – original draft, Writing – review & editing, Data curation, Formal analysis, Methodology, Project administration, Validation, Visualization. MV: Data curation, Investigation, Software, Visualization, Writing – review & editing. SG: Data curation, Software, Visualization, Writing – review & editing. AC: Investigation, Resources, Writing – review & editing. LW: Investigation, Resources, Writing – review & editing. ST: Investigation, Resources, Writing – review & editing. JD: Investigation, Resources, Writing – review & editing. PK: Funding acquisition, Investigation, Project administration, Resources, Supervision, Validation, Writing – review & editing. CH: Conceptualization, Data curation, Formal analysis, Funding acquisition, Investigation, Methodology, Project administration, Resources, Software, Supervision, Validation, Visualization, Writing – original draft, Writing – review & editing.

## Footnotes

^1^ https://surfer.nmr.mgh.harvard.edu/
