# Supplementary material for: Decoding executed and imagined grasping movements from distributed non-motor brain areas using a Riemannian decoder
Source: Front Neurosci. 2023 Nov 23;17:1283491. doi: 10.3389/fnins.2023.1283491 (PMC10701391; doi:10.3389/fnins.2023.1283491)
Supplement: Supplementary file 1 [file Data_Sheet_1.pdf]

#	Age	Sex	Sample rate	Electrodes	Contacts		Noise		Motor
					Executed	Imagined	Executed	Imagined	
1	16	M	2048	14	116	116	8	8	10
2	47	M	1024	11	110	108	20	22	0
3	52	M	1024	6	54	52	65	67	9
4	22	F	1024	5	42	44	85	83	0
5	20	F	1024	11	106	105	13	14	6
6	40	M	1024	12	117	117	13	13	0
7	55	F	1024	12	108	105	15	18	8
8	34	M	1024	11	108	106	17	19	3

Supplementary Table 1: Participants and their electrode configurations. 'Contacts' denotes the amount of contacts after noise and motor removal. 'Motor' denotes the amount of contacts located in an area surrounding the central sulcus.

Supplementary data 1: List of removed areas surrounding the central sulcus

- ctx-rh-paracentral
- ctx-rh-precentral
- ctx-rh-postcentral
- wm-lh-paracentral
- wm-lh-postcentral
- wm-lh-precentral
- wm-rh-paracentral
- wm-rh-postcentral
- wm-rh-precentral
- ctx-lh-G_paracentral
- ctx-lh-G_postcentral
- ctx-lh-G_precentral
- ctx-lh-G_subcentralctx-lh-S_central
- ctx-lh-S_paracentral
- ctx-lh-S_postcentral
- ctx-lh-S_precentral-Inferior-part
- ctx-lh-S_precentral-Superior-part
- ctx-lh-S_subcentral_ant
- ctx-lh-S_subcentral_post
- ctx-rh-G_paracentral
- ctx-rh-G_postcentral
- ctx-rh-G_precentral
- ctx-rh-G_subcentralctx-rh-S_central
- ctx-rh-S_paracentral
- ctx-rh-S_postcentral
- ctx-rh-S_precentral-Inferior-part
- ctx-rh-S_precentral-Superior-part
- ctx-rh-S_subcentral_ant
- ctx-rh-S_subcentral_post
- wm-lh-G_paracentral
- wm-lh-G_postcentral
- wm-lh-G_precentral
- wm-lh-G_subcentral
- wm-lh-S_central
- wm-lh-S_paracentral
- wm-lh-S_postcentral
- wm-lh-S_precentral-Inferior-part
- wm-lh-S_precentral-Superior-part
- wm-lh-S_subcentral_ant
- wm-lh-S_subcentral_post
- wm-rh-G_postcentral
- wm-rh-G_precentral
- wm-rh-G_subcentral
- wm-rh-S_central
- wm-rh-S_paracentral
- wm-rh-S_postcentral
- wm-rh-S_precentral-Inferior-part
- wm-rh-S_precentral-Superior-part
- wm-rh-S_subcentral_ant
- wm-rh-S_subcentral_post
- ctx_lh_G_and_S_paracentral
- ctx_lh_G_and_S_subcentral
- ctx_lh_G_postcentral
- ctx_lh_G_precentral
- ctx_lh_S_central
- ctx_lh_S_postcentral
- ctx_lh_S_precentral-inf-part
- ctx_lh_S_precentral-sup-part
- ctx_rh_G_and_S_paracentral
- ctx_rh_G_and_S_subcentral
- ctx_rh_G_postcentral
- ctx_rh_G_precentral
- ctx_rh_S_central
- ctx_rh_S_postcentral
- ctx_rh_S_precentral-inf-part
- ctx_rh_S_precentral-sup-part
- wm_lh_G_and_S_paracentral
- wm_lh_G_and_S_subcentral
- wm_lh_G_postcentral
- wm_lh_G_precentral
- wm_lh_S_central
- wm_lh_S_postcentral
- wm_lh_S_precentral-inf-part
- wm_lh_S_precentral-sup-part
- wm_rh_G_and_S_paracentral
- wm_rh_G_and_S_subcentral
- wm_rh_G_postcentral
- wm_rh_G_precentral
- wm_rh_S_central
- wm_rh_S_postcentral
- wm_rh_S_precentral-inf-part
- wm_rh_S_precentral-sup-part
- ctx-lh-primary-motor
- ctx-lh-premotor
- ctx-rh-primary-motor
- ctx-rh-premotor

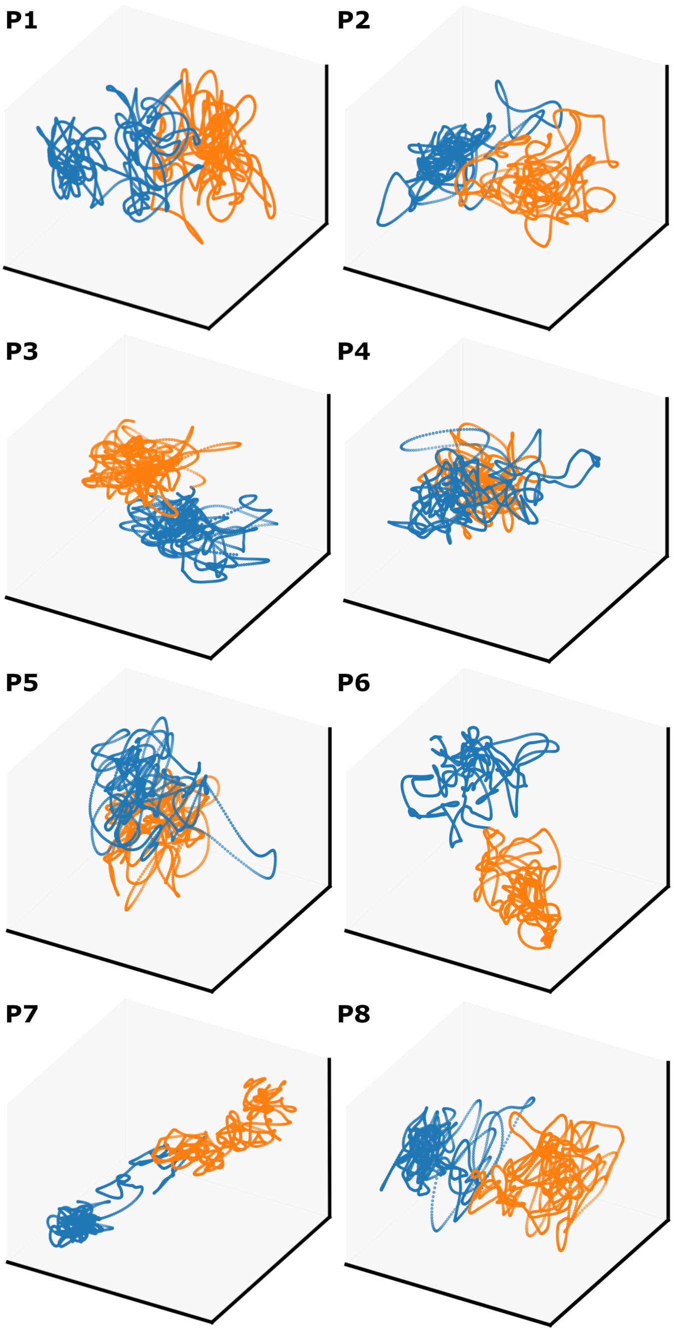

Supplementary Figure 1: Unsmoothed trajectories in components space. Calculated from beta activity in executed movements. The trajectories of P8 are the same as in Figure 1c.

	3	5	10	15	20	25	30	35	40	45	50
Exec - Beta	0.98	0.71	0.83	0.89	0.84	0.90	0.98	0.98	1.00	0.95	0.94
Exec - High-gamma	0.86	0.79	0.93	0.86	0.95	0.75	0.91	0.89	0.92	1.00	0.97
Exec - Beta + High-gamma	0.83	0.73	0.87	0.76	0.82	0.75	0.63	0.91	0.91	0.30	0.72
Imag - Beta	0.65	0.47	0.44	0.88	0.74	0.84	0.84	0.66	0.87	0.78	0.85
Imag - High-gamma	0.80	0.97	0.75	0.77	0.98	0.92	0.93	0.82	0.79	0.80	0.76
Imag - Beta + High-gamma	0.85	0.78	0.79	0.75	0.80	0.79	0.75	0.75	0.84	0.83	0.88

Supplementary Table 2: P-values for significance tests between the performance using all electrodes and the performance without motor cortical areas. None of the tests were significant, meaning that the null hypothesis that both groups come from the same distribution cannot be rejected.

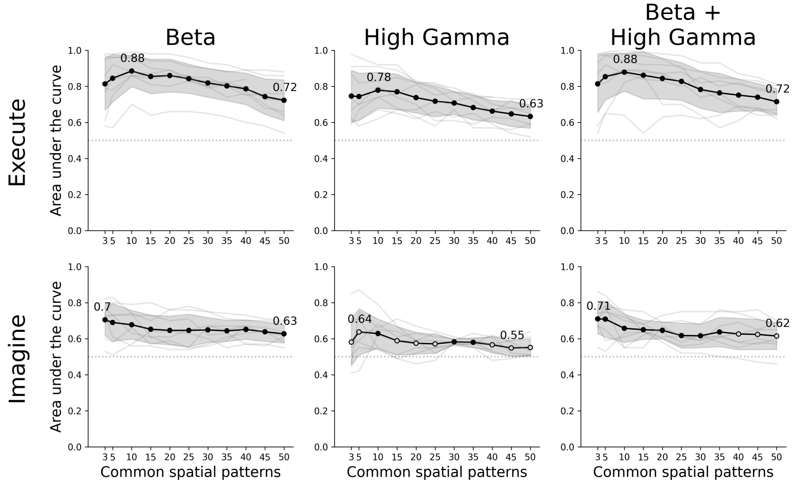

Supplementary Figure 2: Decoding performance for a common spatial pattern (CSP) and linear discriminant analysis (LDA) decoder. Compared to the Riemannian decoder, the CSP-LDA decoder performs better with fewer spatial filters ($< \pm 25$), while the Riemannian decoder performs better with more components ($> \pm 25$).

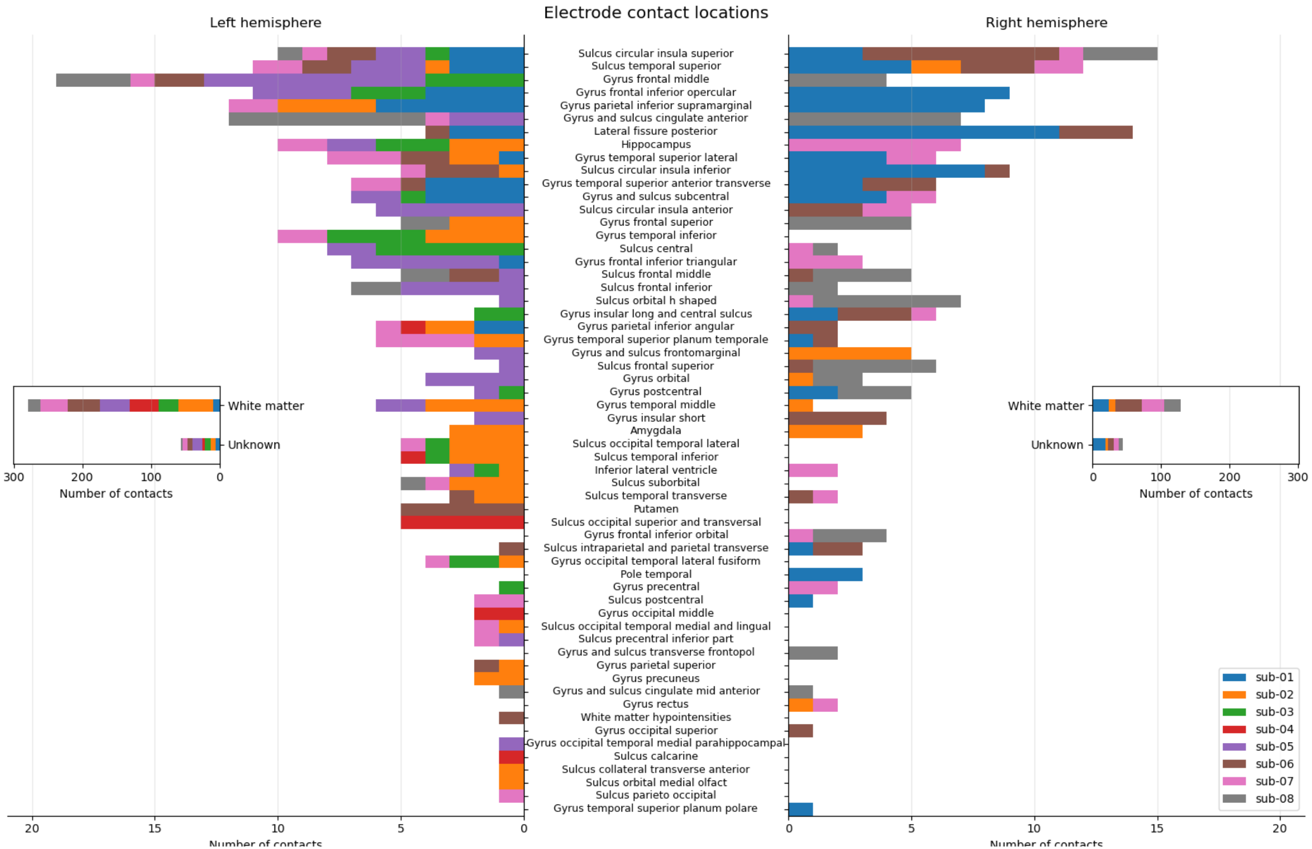

Supplementary Figure 3: Captured areas by all contacts per hemisphere. The insets shows all contacts in white matter and labeled unknown. Note that the size of the X-axis on the insets is much larger than the main figure.
